# Learning under constraints: a theoretical framework for comparing resource-constrained learning in biological and artificial systems

**DOI:** 10.3389/fncom.2026.1636604

**Published:** 2026-06-17

**Authors:** Dmytro Kucherov, Serge Dolgikh, Serhii Podelskyi

**Affiliations:** 1 Department of Intelligent Cybernetic Systems, State University “Kyiv Aviation Institute”, Kyiv, Ukraine; 2 Department of Artificial Intelligence, Lviv Polytechnic National University, Lviv, Ukraine

**Keywords:** artificial cognitive systems, biological cognitive systems, constrained optimization, evolutionary intelligence, resource-constrained cognition

## Abstract

Learning processes, cognitive architectures, available resources, and methods for sampling the environment and generating intelligent responses in complex sensory domains can differ significantly between natural and artificial systems. In this work, we present theoretical and modeling-based analysis of early-stage learning under resource constraints, comparing biological intelligence with a class of freely evolving, weakly constrained artificial systems (FEW), focusing on essential resource constraints such as computational capacity, memory, and energy. We develop quantitative models of sensory exploration and learning under strong and weak resource constraints, formalizing how limitations in energy, memory, and computational capacity shape sampling strategies and learning dynamics. For biologically constrained systems, we show that steep anisotropy in the cognitive cost gradient induces prioritized, depth-oriented exploration within limited sensory regions, leading to robust and resource-efficient learning. In contrast, we demonstrate that FEW systems, despite access to abundant resources, face a paradox of unconstrained learning: in the absence of intrinsic prioritization and evaluative feedback, uniform or random sampling leads to inefficient exploration of the sensory domain. To examine this challenge, we introduce a comparative framework for evaluating sensory traversal strategies and show that no single strategy dominates across prioritization accuracy, robustness, and resource efficiency. Instead, our analysis suggests a meta-strategy approach, in which adaptive selection among exploration strategies optimizes empirical success while preserving empirical accountability required for adaptive optimization. These results clarify the functional role of constraints in biological learning and provide principled guidance for the design of next-generation artificial learning systems operating in complex sensory environments.

## 1 Introduction

Recent years have witnessed remarkable advances in intelligent technologies, with systems now capable of modeling and engaging in intelligent discourse across multiple media and sensory domains, including the integration of several sensory inputs simultaneously. However, despite this progress, there remain significant reservations about whether these developments truly represent steps toward general intelligence that characterizes their biological counterparts. As many have argued, general intelligence should entail the capacity for independent exploration of sensory environments, along with mechanisms for optimizing adaptation and selecting intelligent functions, resources, and cognitive architectures. To address these essential challenges, the question, how intelligent learners can employ limited resources available to them in the optimum way to maximize the learning results can be central.

Any intelligent system, whether biological or artificial, operates under constraints imposed by the resources required to sustain perception, learning, and action. These include memory, computational capacity, energy, and material or physical substrates. While such constraints are fundamental to all intelligent systems, their nature, severity, and functional consequences differ markedly between natural and artificial forms of intelligence.

In biological systems, strong and inescapable constraints are tightly coupled to survival and persistence, enforcing continuous empirical accountability: learning strategies must succeed under limited resources or fail outright (wherefore, *empirical success* is understood as the degree to which a system’s learning and action improve the stability and adequacy of its responses across the sensory environment it can effectively sample).

In contrast, many contemporary artificial systems operate under comparatively weak or externally managed constraints, allowing learning processes to proceed without direct pressure to prioritize, adapt, or economize in response to empirical success or failure. This asymmetry raises a central challenge for the development of more autonomous artificial learners: how resource constraints and empirical feedback jointly shape effective learning strategies.

In this study, we attempted to examine these differences by introducing a comparative functional framework and developing quantitative models that characterize how essential constraints influence sensory sampling, prioritization, and adaptive learning in biological and artificial systems.

## 2 Background and related work

Learning in natural intelligent systems almost always unfolds under strong physical and cognitive constraints, which fundamentally shape their developmental and adaptive strategies. Physical constraints include limited metabolic energy, restricted neural transmission speeds, and finite sensing capabilities (Niven and Laughlin, 2008; Lennie, 2003). Cognitive constraints encompass bounded memory, limited working memory capacity, and temporal constraints on decision-making (Cowan, 2010; Lieder and Griffiths, 2020). These constraints are not incidental but appear to be constitutive of biological cognition, influencing everything from perception and attention to learning and long-term planning.

In contrast, artificial systems including modern deep learning-based models are typically deployed in resource-rich training environments, often optimized for throughput rather than ecological efficiency. While most contemporary artificial learning systems achieve strong empirical performance under conditions of data and computational abundance, resource constraints such as energy, latency, and memory are typically treated as external engineering considerations rather than internally represented variables shaping learning dynamics. Although emerging neuromorphic and edge-aware approaches explicitly operate under such constraints (Indiveri and Liu, 2015; Shi et al., 2016), they are primarily optimized at the hardware level and rarely integrate resource awareness into adaptive exploration or strategy selection. In contrast to biological systems, where resource limitations are intrinsically embedded and continuously integrated into perception, action, and learning constraints in artificial systems are usually predefined, static, or architecturally imposed, rather than learned and dynamically traded off against empirical success. From this perspective, recent constraint-aware architectures can be seen as promising but partial approximations of mechanisms that are foundational in biological cognition, reinforcing rather than undermining the central claim of this work: that internally represented and actively negotiated resource constraints play a constitutive role in adaptive learning strategies.

In Shi et al. (2016), authors proposed the concept of resource-rationality in human cognition, arguing that it approximates optimal behavior under constraints of time, energy, and memory, and suggested this framework as a basis for building efficient artificial systems. This distinction has further motivated comparative research into the fundamental differences between human and artificial intelligence (Anderson, 2007). Whereas biological intelligence evolves under pressure to develop adaptive strategies within tight constraints, artificial systems, in many cases freed from such limitations, may adopt fundamentally different cognitive pathways.

Cognitive architectures such as Soar and ACT-R have been developed to emulate human-like cognition by integrating symbolic reasoning with procedural learning (Anderson et al., 2004; Clark, 1997). These architectures aim to capture the adaptive and resource-aware strategies observed in human cognition. However, they remain structurally distinct from many modern artificial systems, which typically operate without the physical and cognitive constraints inherent to biological agents. Moreover, the lack of physical embodiment in many artificial systems further accentuates these differences. Theories of embodied cognition emphasize that interaction with the environment is essential for the emergence of key cognitive functions (Korteling et al., 2021). Artificial systems that are not sensorimotor-grounded may thus evolve alternative strategies effective within their own context, but cognitively divergent from natural intelligence.

To address this asymmetry, the framework of resource-rational analysis has been proposed as a way to model cognition as near-optimal or optimum-seeking under bounded resources (Miconi et al., 2018; Dolgikh, 2024). This approach seeks to explain not only cognitive efficiency but also human heuristics and biases as emergent from adaptive trade-offs.

Other studies have drawn on biological principles to inform artificial learning architectures. For example, local learning and plasticity mechanisms inspired by neurophysiology have been used to improve learning adaptability and continuity (Anderson, 2007; Zenke et al., 2017). In parallel, models of sensorimotor grounding and intrinsic motivation have been advanced to address the absence of internally meaningful learning signals in artificial agents (Kiverstein et al., 2022). Recent studies have emphasized architectural inductive biases, such as modularity and compositionality as essential for achieving more flexible and generalizable learning, in closer alignment with the structural features of human cognition (Goyal and Bengio, 2021) whereas (Dolgikh, 2024) examined the processes of self-organization of advanced cognitive functions in an evolutionary process driven by empirical optimization objective under resource constraints.

Recent research has highlighted the significance of information-theoretic approaches in understanding cognitive systems operating under resource constraints. For instance, Béna and Goodman (2025) emphasized leveraging information-theoretic principles to bridge human and artificial cognition, focusing on how information storage and processing limitations shape cognitive functions and neural representations. Additionally, studies have explored how resource constraints drive the emergence of functional specialization in artificial neural networks. For example, it has been shown that limiting network capacity, such as bottlenecking parameters or restricting energy usage can lead to the spontaneous development of specialized sub-modules that mirror hierarchical and modular structures found in biological cognition (Achille and Soatto, 2018; Bengio et al., 2015). These findings suggest that functional differentiation may not require explicit architectural design, but can emerge naturally under pressure to optimize representational efficiency. Such insights reinforce the idea that resource limitations are not merely obstacles, but structural forces that shape learning strategies, internal organization, and cognitive development across both artificial and biological systems. Our work is closely aligned with this perspective, emphasizing the role of constraints not just in limiting performance, but in directing the structure and dynamics of learning itself.

While biological and artificial systems differ fundamentally in structure, embodiment, and constraints, an integrative perspective may yield deeper insights into adaptive intelligence. Lessons from biological cognition: such as context-sensitive sampling, plasticity, and energy-efficient representation can inform the design of more robust and flexible artificial systems (Hassabis et al., 2017; Lake et al., 2017). Conversely, formal modeling and simulation in artificial settings provide a controlled framework to test hypotheses about learning under constraints, offering valuable tools for cognitive science and neuroscience (Eliasmith, 2013). Bridging these domains encourages the development of hybrid models that combine biologically inspired mechanisms with computational scalability, promoting both theoretical understanding and practical advances.

In summary, prior work provides partial accounts of constrained learning, sensory exploration, and adaptive behavior, but can be strengthened by a comparative framework that treats resource constraints, prioritization mechanisms, and empirical success as co-determining factors of cognitive evolution. In particular, there is little work addressing (i) how weakly constrained artificial systems differ qualitatively from biologically constrained learners, (ii) how exploration strategies can be evaluated under persistence-based success criteria, and (iii) how adaptation-oriented cognitive organisations and strategies may facilitate empirical success.

In this work, we attempt to address these gaps by combining a comparative functional analysis of the role and impact of essential constraints: both cognitive and physical on the formation, adaptation, and selection of successful learning strategies, where empirical success is evaluated in terms of empirical performance relative to the learner’s essential or existential objective, such as survival-relevant competence or robust generalization across the domain of relevant inputs. The analysis is structured in three closely interrelated research questions:

*How do the nature and severity of essential resource constraints influence the organization and effectiveness of sensory exploration and learning strategies in biological versus artificial learning systems*?

*How do different constraint regimes affect the balance between learning depth (robustness and resolution) and learning span (conceptual breadth) during early-stage learning*? and

*Under what conditions can adaptive or meta-level mechanisms for strategy selection improve empirical success in weakly constrained artificial learners, compared to fixed exploration strategies*?

These questions are explored using comparative analysis by identified functional criteria and development of quantitative models designed to capture the interaction between resource availability, exploration dynamics, and empirical feedback.

## 3 Theoretical framework

In this study, we employ a comparative functional analysis framework to examine the influence of resource constraints on learning mechanisms in biological and artificial intelligent systems. In the parallel comparative analysis we use in this work, we will attempt to compare characteristic sensory environments, essential constraints and certain basic learning processes in a strongly-constrained biological system (a reference example can be a mammal child in the early age and stage of cognitive development) and of proposed next stage artificial intelligent system that can be characterized by cognitive resources including memory, processing power (compute), energy and so on a similar level with the state-of-the-art intelligent systems of today or near future; plus an ability and a certain freedom to explore its sensory environment, select and construct responses and adapt, modify or enhance its cognitive configuration to pursue the optimum empirical success as determined and guided by its essential objective/imperative.

By contrasting these two paradigms: biological learning shaped by necessity and artificial learning enabled by abundance we aim to identify principles that could improve the efficiency, adaptability, and cognitive realism of future artificial systems.

### 3.1 Essential constraints in natural and artificial learning

The constraints that we will consider in this work can be of two main types: cognitive and physical. Intelligent systems of either nature, biological or artificial operate within the scope of physical laws of nature and are constrained by the resources available to them in the process of interaction with the sensory environment, interpretation of sensory stimuli, construction of intelligent responses, storing, retrieving and utilizing information, response and other models and knowledge.

The constraints and accordingly, their constraints in an intelligent system regardless of its nature and origin can be broadly classified into cognitive, physical, and structural/architectural categories:

Cognitive resources include memory capacity, computational power, and elements related to information processing such as the availability of synapses (in biological systems), and limitations in processing methods, functions, and representational capabilities. These constraints influence not only how much information can be processed and stored, but also the form in which knowledge can be represented and generalized. In many systems, limitations in representational depth or flexibility can significantly shape learning outcomes.

Physical resources refer primarily to the energy and material inputs required to support the entire learning cycle, including sensing, computation, and actuation. This category also includes, importantly, temporal constraints, such as processing latency and response time, which place hard limits on real-time behavior and influence how efficiently a system can adapt to dynamic environments.

Structural and architectural constraints involve the underlying organization of the system’s components, neural, algorithmic, or hardware-based and the extent to which this structure is fixed or modifiable. These constraints determine how flexibly the system can reorganize itself in response to learning or environmental changes. Biological systems, for example, often exhibit high degrees of plasticity and modularity, while most artificial systems rely on rigid, pre-defined architectures with limited capacity for self-restructuring.

### 3.2 Biological learning

Biological intelligent systems demonstrate a remarkable capacity for efficient and adaptive learning under severe resource limitations. The human brain, for example, operates on approximately 20 watts of energy (Madhavan, 2023), yet is capable of supporting complex cognitive functions such as abstract reasoning, sensory integration, long-term memory, and behavioral adaptation. This energy efficiency is achieved not through brute-force computation, but through tightly optimized neural mechanisms such as sparse coding, synaptic plasticity, and task-specific attentional modulation.

One of the defining features of biological learning is its intrinsic prioritization of sensory stimuli based on relevance to survival and task demands. Rather than attempting to process all incoming information uniformly, biological agents employ selective attention (Posner and Petersen, 1990), guided by internal goals, motivational rewards (Berridge and Robinson, 1998) and contextual relevance, to allocate limited cognitive resources effectively and efficiently. Learning unfolds in a continual, incremental manner, supported by neurophysiological mechanisms such as memory consolidation during sleep and homeostatic plasticity (Walker and Stickgold, 2004; Turrigiano, 2012). These mechanisms allow organisms to integrate new information while preserving previously acquired knowledge, avoiding the phenomenon of catastrophic forgetting that plagues many artificial systems (French, 1999).

Furthermore, biological intelligence is inherently goal-directed and self-regulating. Organisms evaluate their actions through endogenous feedback loops tied to emotional valence, reward, and interoceptive states (Rolls, 2000; Schultz et al., 1997; Craig, 2002), enabling them to adapt flexibly to novel and uncertain environments. Such properties underscore a form of context-sensitive intelligence that is deeply embedded in the constraints of the physical body and its ecological niche (Clark, 1998).

It is also important to outline additional essential characteristics of early-stage learning in biological systems, particularly in more advanced species as mammals and others. First, such systems are capable of learning from small and selectively focused samplings of their sensory environment. For example, a human or mammalian infant may encounter only tens of thousands of critical visual stimuli in the early months, yet these inputs are often highly relevant and meaning-dense, enabling selective focus learning. A conservative estimate, on the order of 100 essential visual impressions per day over 100 days suggests that effective learning can take place with dramatically less data than what artificial systems typically require.

Second, learning in biological agents unfolds through extended trial-and-error routines, often over many iterations. This process fosters resilience to distortion: instead of rigid or idealized representations, biological learners form flexible, fuzzy models that reliably identify essential entities even when inputs are noisy, distorted, altered, or incomplete (e.g., learning strategies fostering robust recognition of sensory inputs seen in infant-directed speech or “baby talk” (Nencheva and Lew-Williams, 2022)).

Third, biological systems can demonstrate non-deterministic behavior in response to uncertain yet critical stimuli. When no clear or pre-established response is available, these systems can favor tentative action guided by the rationale “better try and fail than fail to try” which aligns with principles such as the Boldness heuristic or Pascal’s Wager (Gigerenzer et al., 2011).

Fourth, learning is fundamentally interactive and embodied. Infants acquire knowledge by engaging directly with their environments, probing sensory boundaries and testing outcomes through active exploration (Altmann et al., 2025). This interactive modality contributes to the early formation of context-sensitive responses and a high degree of response differentiation, even in the presence of limited input.

Finally, biological learning proceeds via anisotropic, priority-driven expansion of the sensory domain. Initial learning is concentrated in a narrow “core” domain, often anchored to survival-relevant features. Expansion of understanding proceeds outward based on perceived or empirically confirmed relevance, and critically, this learning can occur without the need for massive resources upfront. Instead, it unfolds iteratively, with gradual enlargement of both cognitive scope and underlying neurophysiological resources. This reflects a fundamentally resource-efficient adaptation strategy, guided by existential imperatives rather than externally imposed goals.

Consequently, the perceived sensory scope of the biological learner is highly stratified, layered, and prioritized. In early stages, learning concentrates on relatively small subregions of its sensory domain that hold the highest existential relevance, i.e., those most critical for immediate survival and functional development. Sampling is selectively focused: effective responses to the most salient and proximal stimuli within the “interactive shell” of the sensory environment are developed first, forming a robust foundation for subsequent learning. From these initial phases, robustness and resilience are actively built enabling reliable recognition and appropriate responses even when sensory inputs are strongly distorted or uncertain.

The prioritization of learning is guided by internal assessments of existential relevance, aligned with the organism’s survival and developmental goals. As a result, the system achieves both high empirical success across the conceptual landscape of the learned domain (lateral generalization) and depth-wise robustness to sensory variation and distortion. Crucially, this is accomplished with strong efficiency: learning proceeds incrementally and adaptively under tight constraints on essential cognitive and physical resources such as memory, processing capacity and energy. Thus, it is both effective, achieving high level of empirical success and efficient, operating under strong constraints of essential cognitive and physical resources.

### 3.3 Artificial learning

We do not aim to provide an exhaustive review of the current state of artificial intelligence, which has been covered in recent surveys (Marcus and Davis, 2019; Russell and Norvig, 2020), among others, and instead focus on aspects most relevant to learning under resource constraints. Instead, we focus on a narrower selection of developments directly relevant to our work: the ability of intelligent systems to learn, defined here as achieving high and consistent empirical success across a sensory domain through interactions with the environment while operating under constraints on cognitive and physical resources.

Artificial intelligent systems, particularly those built on deep learning and large-scale architectures, have demonstrated remarkable success in tasks requiring high-dimensional pattern recognition, long-range dependencies, and the handling of data-rich environments. Notable systems such as GPT-3, PaLM, AlphaZero, and CLIP have set new benchmarks in natural language processing, strategic gameplay, and image-text alignment. These systems excel in environments with abundant data, but their performance often hinges on access to vast computational and data resources, highlighting the critical role of resource constraints in their learning capabilities.

These systems were designed to leverage vast computational and memory resources, operating in environments of data abundance and near-unlimited processing power. Their learning paradigms are heavily reliant on externally defined objective functions, such as loss minimization over large datasets, rather than on autonomous goal formation. While this approach enables high throughput and strong generalization within narrowly defined problem domains, it often leads to issues such as rigidity, overfitting, and poor transferability across tasks (Zhang et al., 2021).

Moreover, most current artificial systems lack internal representations of resource constraints. As a result, they do not adapt their learning behavior based on feedback related to energy consumption, processing time, or memory usage. These systems also suffer from catastrophic forgetting, requiring continual retraining or architectural expansion to incorporate new tasks without overwriting prior knowledge (Kirkpatrick et al., 2017). Furthermore, the computational efficiency and environmental costs of training large models raise significant concerns about sustainability and accessibility (Strubell et al., 2019).

Other notable cognitive characteristics of artificial intelligent systems at the current state of technology include:

***Limited structural variability, flexibility, plasticity***: These systems exhibit a much narrower range of sampling and structural autonomy compared to natural intelligence, which allows for more flexible adaptation and exploration.

***Large upfront resource requirements***: Current AI systems demand massive computational power, memory, and energy resources for training, often requiring significant infrastructure just to complete the learning process.

***Massive data sampling***: These systems require extensive and highly curated datasets to train effectively. For example, large language models (LLMs) often rely on datasets that would take centuries of sensory data to replicate in a biological system, raising practical concerns about data sufficiency and representativeness^1^.

Notably, such conditions may be impractical or even infeasible for natural, biological systems, which are typically strongly constrained in both physical and cognitive resources.

As a result, the sensory scope of current stage artificial systems differs significantly from that of biological systems in that it is not prioritized or differentiated. These systems treat the entire sensory input space as a unified block of data rather than distinguishing between different sensory signals based on relevance or context. This lack of differentiation undermines the ability to construct adaptive, context-specific response-forming mechanisms. Furthermore, most current systems lack robust feedback loops to verify empirical success, optimize information models, or refine response functions. As a result, their resilience to distortion is limited, and any adaptation to environmental changes is largely constrained by the external sampling mechanisms provided by the system’s designers.

To summarize, while artificial systems excel at imitating the data space they are trained on, achieving very good to excellent performance within that domain, they struggle when faced with regions outside the training sample, where they are less effective due to insufficient sampling and lack of flexibility. Despite their extraordinary scale, precision, and success in specific tasks, these systems often lack the flexibility, resource efficiency and contextual awareness characteristic of natural intelligence. The fundamental difference between natural biological systems and the current generation of artificial systems lies in their operating conditions. While both can achieve comparable levels of success in specific tasks, under conditions we will discuss further, the artificial systems function under very different constraints regarding the essential cognitive and physical resources needed for empirical success in the sensory environments they are designed to learn.

### 3.4 Comparative analysis of biological and artificial learning models

To summarize the analysis of distinct characteristics of biological and current state artificial learning, we conducted a structured comparison along the core dimensions: *resource awareness*, including energy consumption, memory capacity, and temporal efficiency; *structural plasticity*, reflecting the system’s ability to self-regulate learning and define internal goals; *sampling autonomy*, focusing on autonomy, selectivity and stimulus prioritization; and *empirical feedback* for evaluating empirical success and guiding behavioral adaptation. In addition, we considered the system’s capacity to form and update empirical goals (*goal-directed behavior*), as this capability underpins priority-based sampling and the effective learning of stimuli relevant to system persistence.

These dimensions were selected because each bears directly and independently on the system’s capacity to evolve its cognitive configuration in response to probabilistic regularities in sensory feedback. Specifically, they capture complementary aspects of adaptive learning: how resources bound learning dynamics, how goals and internal structure are generated, how sensory information is selectively acquired, and how outcomes are evaluated and integrated over time. Importantly, these factors are treated as largely orthogonal: variation along one dimension does not trivially or necessarily induce corresponding variation along the others. For example, increased resource availability does not guarantee improved prioritization accuracy, nor does structural autonomy alone imply efficient sampling or robust feedback evaluation. This separation allows for clearer attribution of observed differences in learning behavior and empirical performance and reflects essential mechanisms that influence how learning unfolds under constraints.

We employed a categorical (interval-scale) evaluation methodology designed to reflect the functional impact of each characteristic on the system’s decision-making process, including decisions affecting long-term persistence and response to critical stimuli. Rather than treating capacities as abstract traits, each factor was ranked according to its expected influence on empirical outcomes under resource-constrained learning. The evaluation categories were defined as: *minimal/vanishing, small, moderate, significant,* and *critical*, corresponding to increasing degrees of causal relevance in adaptive decision processes. These rankings were derived from a structured synthesis of the relevant literature and conceptual analysis of system capabilities. Although this approach may fall short of precise quantitative measurement, it is adequate for our purpose of isolating structurally significant differences between learning processes and provides a natural basis for future quantitative development.

**Figure 1:**
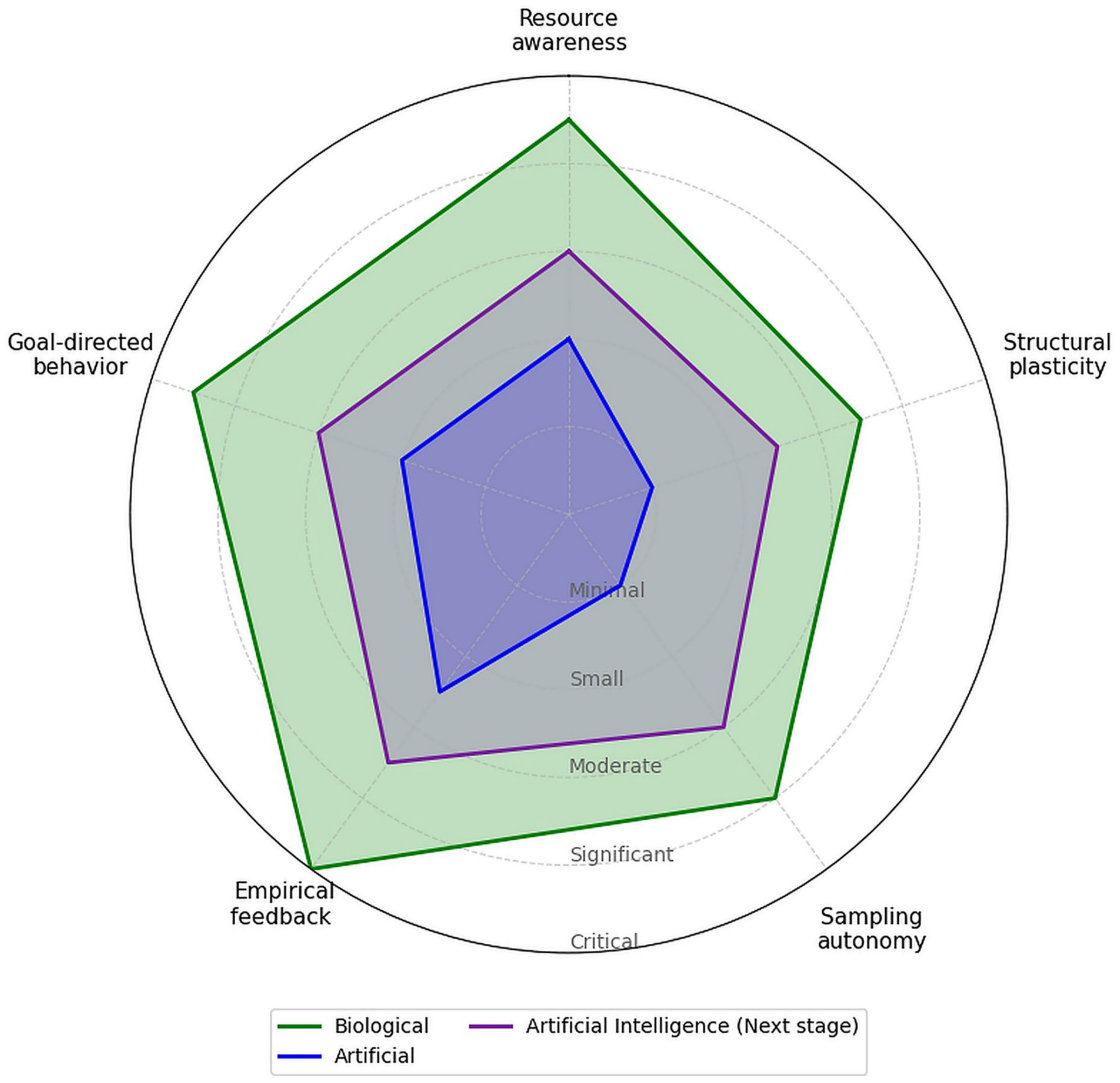
Comparative evaluation of biological and artificial learners (current state) over key learning dimensions.

Figure 1 depicts a visual synthesis of the comparative profiles of biological and current-stage artificial cognitive systems across the selected characteristics, represented as a star chart. The figure is intended to provide a high-level visualization of the contrast between the two classes of systems. A more detailed evaluation and justification of the characteristic values is provided in Appendix Table B1.

Natural (biological) systems show superior performance in resource awareness, goal-directed behavior, and structural plasticity, the traits closely tied to their self-regulating and context-aware character of their embedding into interactive niche. In contrast, artificial systems dominate in availability of cognitive resources but fall behind in internal motivation, feedback-driven adaptation, and contextual learning. This visualization reinforces the central argument: while the current cohort of artificial systems has proven effective in specific cognitive context, they lack the intrinsic adaptability and constraint-aware learning mechanisms inherent to natural intelligence.

This comparison first, highlights an essential difference in the learning characteristics between advanced natural intelligence and current state artificial systems; secondly, it suggests a fundamental trade-off: biological systems forgo raw speed in favor of efficiency, resilience, and autonomous regulation, whereas artificial systems prioritize speed and precision, often at the expense of adaptability, contextual sensitivity, and energetic economy. Crucially, the lack of internal resource awareness and flexible, self-generated goals in current artificial systems constrains their ability to operate effectively in open-ended, unpredictable environments.

## 4 Exploration strategies in natural and artificial learning

To illustrate and further substantiate the observations made in this study, we introduce a set of quantitative models that characterize the learning processes of both biological and artificial intelligent systems under explicit cognitive and physical resource constraints. These models serve as a framework for comparative analysis of learning strategies, aiming to identify essential structural and behavioral differences. Through this analysis, we seek not only to highlight the current limitations of advanced artificial systems, but also to explore potential directions for addressing these limitations and guiding the development of more adaptable, efficient, and context-aware intelligence.

### 4.1 Early-stage natural intelligence

We begin by considering the learning scenario of a strongly constrained natural intelligent agent, such as an infant of an advanced mammalian species. This type of learner is characterized by:

- Severe limitations on cognitive and physical resources, including memory, processing capacity, energy, and materials.
- High flexibility and plasticity in adapting to its immediate sensory environment.
- An active, interaction-driven learning process, shaped by continuous engagement with the surrounding sensory stimuli.

Given the strongly constrained nature of this scenario, it is appropriate to frame the learning process as a constrained optimization problem, as examined in Dolgikh (2024) and Smith and Thelen (2003). This formulation provides a useful approach for examining how such a system allocates its limited resources to maximize learning outcomes within its local sensory environment.

#### 4.1.1 Utility-guided learning under resource constraints

To illustrate the learning mechanisms of biological intelligent systems operating under strict resource constraints, a utility-based model of prioritization of sensory inputs and allocation of limited cognitive resources can be proposed. The model is based on the assumption that a learner aims to maximize empirical learning success by selectively attending to stimuli that are both existentially significant and feasible to process given current limitations in memory, energy, and computational capacity.

This selective process can be formalized as an optimization problem where the system attempts to extract the highest possible (optimal) persistence-relevant information value from its perceptual environment while operating within resource bounds. The corresponding objective function is expressed as follows:


$${\max\;U{\left(S,R\right)}_{S\in D,\;R\in R_{L}}=\sum_{i=1}^{n}\;\pi (S_{i})\;f(S_{i},R_{i})|}_{R_{L}}\;$$ (1)

Where:

- *S* ∈ *D*: denotes sensory stimuli within the domain *D*, the accessible sensory inputs.
- RL: the region of constrained resources available for execution of cognitive functions of the system.
- *π*(*S*_i_): represents the existential priority of the stimulus *S*_i_, reflecting its survival-critical or motivational relevance (i.e., higher value of the factor relates to the importance of the signal is for survival or development).
- *f*( $S_{i}$ , $\;R_{i}$ ): the processing feasibility and efficiency of stimulus $S_{i}$ given available resources $R_{i}$ , which include memory, computational power, time and other essential resources.
- *U*(*S*, *R*): the empirical utility objective, reflecting the cumulative benefit of attending to a selected subset of stimuli.

The relation in (1) formalizes a core biological principle: strongly constrained resources necessitate prioritized learning. Infants, for example, allocate disproportionate attention to sensory inputs like facial expressions, touch, or voices-stimuli with high existential relevance-and only later expand their focus as cognitive resources grow. This process leads to highly efficient learning trajectories that begin with dense sampling of a small “core” sensory region, followed by iterative outward expansion (Werker and Tees, 1984).

#### 4.1.2 A discrete interval model of early-stage natural learning

Due to the strong material constraints present in early-stage biological learning, effective learning can only occur within a narrow subspace of cognitive variation that aligns with an already partially mastered region of the sensory scope. This constrained optimization approach reflects the developmental realities of early learners, such as human or mammalian infants, who acquire knowledge incrementally and selectively in response to essential environmental stimuli (e.g., caregiver faces, vocal tones, object permanence cues).

In this high-level model, we represent the interaction between an intelligent learner and its sensory environment using a simplified cognitive map of the sensory scope. For an infant-like biological system (hereafter referred to as the *biological infant*), this map is conceptualized as a two-dimensional surface embedded in a three-dimensional space characterized by:

Conceptual span *r*: the lateral range of distinct concepts or categories corresponding to sensory stimuli mastered by the learner.
Cognitive depth *y*: the degree of robustness, resilience, and granularity in the internal model’s representation of those concepts.

(At a later time, we may consider temporal progression of learning success metrics, in the context of uniformity across the sensory domain).

Then a point in this conceptual space identified by the conceptual span and cognitive depth coordinates *x* = (*r*, *y*) can represent both:

- A subregion in the infant’s accessible sensory environment; and
- The corresponding region in its internal cognitive model.

This abstraction allows us to formally describe the development of the learner’s internal information model of the interactive environment (sensory scope) as an expansion in both span and depth, guided by interaction with and feedback from the sensory environment.

Next, for every realistic position in the region of sensory inputs that can be perceived by the learner in his current cognitive state, characterized by the position *x* we define the *cognitive success function C*(*x*) as a certain measure of empirical success of the learner in responding to sensory inputs in the proximity of that position. While a precise quantitative formulation of *C*(*x*) is beyond the scope of this work, it can be understood as a performance indicator tied to the learner’s ability to recognize, model, and respond to stimuli in that subregion.

Based on this, we introduce the resource cost of the cognitive success as the gradient:


$$C_{R}(x)=\frac{\partial C(x)}{\partial R}$$

which describes the marginal cost, in terms of some generalized constrained resource *R* (e.g., memory, energy, computation) required to achieve an incremental improvement in cognitive success at position *x*. This formulation is consistent with an interpretation of early-stage learning as a resource-constrained optimization process (1) (Dolgikh, 2024), where the learner strives to allocate limited resources to maximize cognitive success over its sensory environment.

A key observation relevant to the model introduced here is that learning in the direction of cognitive depth is typically more accessible that is, requires fewer resources per unit of cognitive success than learning in the direction of conceptual span. This can be intuitively justified by considering a small variation δ*x* in the cognitive model space, which may consist of dimensions for conceptual span (δ*r*) and cognitive depth (δ*y*). Then, variation in depth, δ*y*, may yield additional competence either by increasing resolution (where *y* + δ*y* corresponds to a distinguishable but related input) or by improving robustness (where variations are interpreted as acceptable distortions of known inputs). In contrast, an equivalent variation in conceptual span, δ*r*, typically requires the learner to acquire and internalize novel concepts that may differ substantially from the mastered ones. For early-stage learners operating, as noted, under strong resource constraints, the resource cost δ*R* associated with such expansions may be insufficient to support this kind of qualitative leap in conceptual content.

The argument just presented can be summarized as the assumption of *steep spatial anisotropy* of the gradient of the cognitive resource cost function in the directions of conceptual span:


$$|\frac{\partial C_{R}(x)}{\partial r}|\gg |\frac{\partial C_{R}(x)}{\partial y}|$$ (2)

or $|G_{R,y}(x)|\ll |G_{R,r}(x)|$ , where *G*_*R*,*d*_(*x*): the gradient of the cognitive cost function at position *x* in the direction *d*. This assumption indicates that expanding in the spatial direction *r* (i.e., covering more sensory inputs) incurs a significantly higher cost than expanding in the depth direction *y*, elaboration of already mastered conceptual region which allows for a more efficient increase in conceptual resolution or robustness. Such a configuration of the cognitive gradient can be described as “a narrow beam” or “searchlight” learning strategy/developmental model. This observation is supported by work in cognitive load theory and incremental learning, which consistently indicates that deepening existing representations is more resource-efficient than expanding conceptual span through the acquisition of novel categories (Sweller, 1988; Elman, 1993).

In the next step, we recall that the condition of the “biological infant” system with respect to the cognitive resources available to it is not static. As it grows in the physical space concurrently with learning, it can acquire additional cognitive resources. For instance, studies have shown that the frontal lobes, responsible for functions like planning, impulse control, and working memory, undergo substantial development during infancy (Dehaene-Lambertz and Spielke, 2015). This growth is crucial for the maturation of executive functions. Additionally, the hippocampus, integral to memory formation, also develops rapidly in the early years, facilitating the encoding and retrieval of information (Kelly et al., 2024; Giedd et al., 2009).

This process can be modeled by a discrete approximation, where the cognitive resources available to the learner (in this model represented by a single cumulative metric parameter) remain constant for the duration of the current learning interval, incrementing by the appropriate amount in the beginning of the one that follows:

##### 4.1.2.1 Discrete interval model of sensory exploration

The development of the “biological infant” system based on results (1, 2) is modeled as a sequence of learning intervals, denoted *I* = {*i*_1_, …, *i*_k_}. During each interval, the cognitive resources available to the learner, denoted *R*_k_, remain constant. At the boundary between intervals, the total cognitive resources are increased by an increment Δ*R*_k_, such that: *R*_k + 1_ = *R*_k_ + Δ*R*_k_.

This discrete process reflects the gradual relaxation of the resource constraint as the system develops physically and acquires additional cognitive resources (e.g., increased processing capacity in the brain).

In each learning interval *k*, the cognitive state of the learner is described by the approximation of the cognitive success function *C*_k_(*r*, *y*) where, as earlier, (*r*, *y*) represents the spatial (conceptual) span and cognitive depth.

The ranges of *r* and *y* are limited by the available cognitive resources in each iteration, so they are bounded by certain values *r*_k_ and *y*_k_ for the *k*-th learning interval. Thus, the cognitive state during each interval is constrained by the learner’s resource capacity, with both the span and depth expanding progressively as resources increase over time.

##### 4.1.2.2 Spatial anisotropy of the cognitive cost gradient under resource constraints

Recalling the observation on the spatial anisotropy of the cognitive cost gradient under strong resource constraints, one can expect that for the development positions within the *k*-th interval, learning happens predominantly along the direction of cognitive depth:


$$\begin{array}{l} C_{y}(x)=\frac{\partial C(x)}{\partial y}=F(x,R_{k},C_{k}(x));|C_{r}(x)|\\ =\frac{\partial C(x)}{\partial r}\ll |C_{y}(x)|\sim 0 \end{array}$$ (3)

where *F* is a function dependent on cognitive resources and the current state of cognitive success. To further simplify (4), we hypothesize that *F* can be approximated by the form:


F≈βf(y,C(y))C(y),

where f(y,C(y)) : a slowly varying function representing a relatively stable relationship between cognitive depth *y* and learning success.

The functional form reflects the assumption that, under strong resource constraints, improvements in cognitive depth primarily act to reinforce and consolidate existing empirical success, making learning efficiency approximately proportional to the current success level C(y) . Contributions from lateral expansion of conceptual span are assumed to be subdominant in this regime and are therefore neglected at leading order (technically, it can be described by another factor: (γ(r)=1+εg(r)),ε≪1 ). The prefactor f(y,C(y)) captures structural and architectural influences on learning efficiency and is treated as a slowly varying function over the learning intervals considered.

The dynamic thus described can produce an accelerated development/improvement of the learning success in the direction of cognitive depth within the previously learned conceptual region of the sensory scope, *r*_k_. It can be visualized as a “searchlight” type of exploration strategy in the sensory/conceptual neighborhood of the current cognitive state where the learner focuses on refining and deepening its understanding of sensory inputs within a localized conceptual area, progressively enhancing the robustness and resolution of responses in this neighborhood.

##### 4.1.2.3 Expanding the sensory scope due to relaxation of constraints

As formulated earlier, learning proceeds in discrete intervals. During interval $i_{k}$ , exploration in the longitudinal (span) direction is strongly suppressed but possibly, nonzero if 0<g≪1 , while resources are primarily allocated to increasing cognitive depth within the currently accessible region. At the beginning of the next learning interval, *i*_k + 1_ the system may acquire additional increment of cognitive resources $\Delta R_{k}$ enabling an expansion of the explored sensory scope in the longitudinal direction.

This expansion can be modeled as:


$$\Delta r_{k}=G(r_{k},\Delta R_{k})$$

where *G* captures the relationship between the current conceptual reach *r*_k_, incremental expansion of the conceptual region $r_{k+1}=r_{k}+\Delta r_{k}$ and the additional cognitive resources *ΔR*_k​_ that are available in the next learning interval. Importantly, G is assumed to be negligible in the absence of new resources: *G*(*r*, 0) = 0 and to dominate over the suppressed lateral exploration term g when $\Delta R_{k}>0$ .

The resulting effect is a discrete widening of the conceptual “cone” (beam) of the learned sensory domain, allowing the learner to access previously unreachable regions. Learning thus proceeds cyclically: depth is consolidated within a fixed region during interval $i_{k}$ (Equation 4), followed by resource-enabled expansion of conceptual span in interval $i_{k+1}$ .

**Figure 2:**
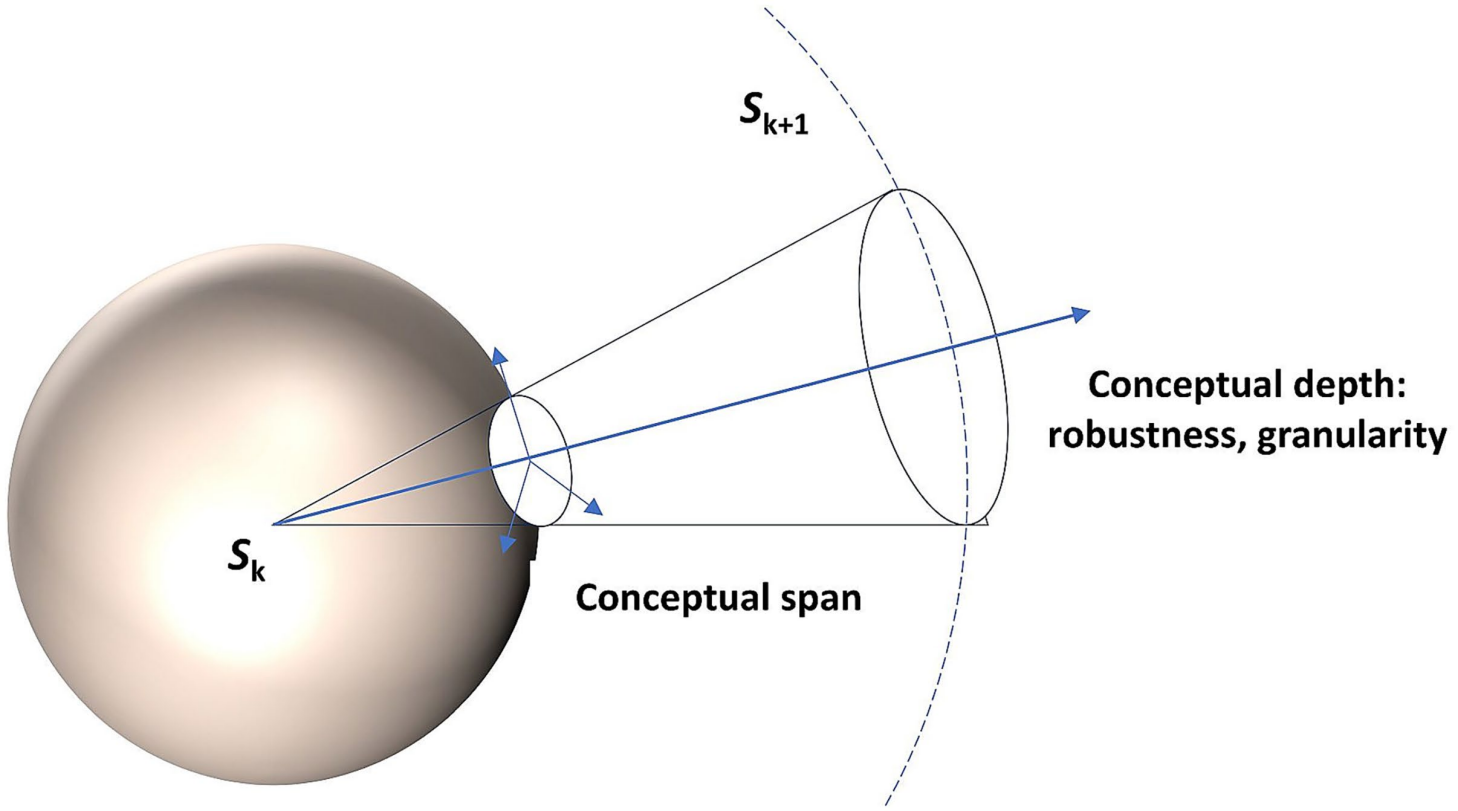
Anisotropic prioritized sensory exploration strategy in early biological learning.

This learning strategy can be visualized as a layered, outward expansion not unlike the peeling of an onion shell in the sensory environment of the learner in the inverse, outward direction. At each step, the learner expands its sensory scope outward, progressively exploring new conceptual regions while strengthening its knowledge and responses within previously learned areas. The result is a structured, gradual and efficient traversal of the sensory space, balancing resource constraints with the need for expanded learning. Thus, the model can be visualized as inverse, inside outward unfolding of an “onion shell”-type traversal of the immediate sensory scope of the early learner (Figure 2).

Finally, as the learner progresses toward maturation, the increments of cognitive resources available per learning interval may change, prompting a shift in the learning strategy. One possible exit occurs when resource increments ΔR exceed the thresholds that previously constrained lateral exploration ( $\Delta R_{k}>r_{C}$ ), enabling simultaneous deepening and expansion of the conceptual scope; in this regime, a narrow-beam strategy is no longer optimal, as broader exploration may yield higher empirical benefit. An alternative exit corresponds to stagnation, where $\Delta R_{k}\to 0$ , leading to vanishing updates in both depth and span and effectively halting learning. These two regimes delimit the operational validity of the anisotropic exploration strategy and motivate adaptive strategy selection as resources and environmental demands evolve. The model of the early stages of cognitive development of a biological intelligent system defined here can be characterized by anisotropic, priority-based probing and sampling within the immediate sensory scope. This approach ensures high resilience to distortion and effective management of uncertainty, while also enabling outward expansion based on the perceived or empirically derived priority of sensory stimuli.

##### 4.1.2.4 Model parameters

The parameters of the model, including the duration of a learning interval τ , the magnitude of resource increment $\Delta R_{k}$ , and related scaling factors, are not treated as universal constants but rather, determined by the cognitive structure (architecture) of the learning system and its embedding in a specific interactive environment. These parameters reflect an implicit trade-off between resource expenditure and empirical benefit: excessively frequent learning cycles with insufficient resource increments may lead to vanishing updates, shallow consolidation, and inefficient use of available resources, while overly coarse updates may delay adaptation. In practice, such parameters are expected to be adaptively regulated to maximize empirical success under system-specific constraints, motivating their exploration through simulation in representative embeddings.

Simulations of the discrete-interval learning model were conducted (Appendix C). They indicate that the proposed formulation is sufficiently flexible to support iterative learning of an interactive environment under varying levels of resource constraints. While the present study uses these simulations primarily for validation and illustration, a more detailed quantitative analysis of parameter regimes and performance trade-offs is deferred to future work.

These conclusions, derived from our discrete anisotropic model of early-stage natural learning, find support in developmental cognitive science. Empirical studies of infant learning consistently show accelerated progress in perception and recognition within initially limited but highly relevant sensory regions, accompanied by increasing tolerance to distortion and uncertainty (Dehaene-Lambertz and Spielke, 2015). This aligns with our assumption of early-stage learning being dominated by depth-oriented optimization within conceptual subregions, supported by gradually increasing cognitive capacity.

### 4.2 Next, stage artificial intelligence: the challenge of autonomous sampling

#### 4.2.1 Next stage artificial learning model (FEW)

We will now introduce a next-stage artificial intelligent system developing just beyond the current horizon of the technology. It can possess cognitive resources on the level of modern state-of-the-art systems but in contrast to them, also has certain freedom to explore their sensory environments independently, including defining sampling methods/strategies of their sensory environments, form, expand and adjust their information models thereof and modify and improve the functions of construction of responses which are verified by empirical success.

There are two substantial arguments for this hypothesis. First, current state-of-the-art artificial systems exhibit a fundamentally static approach to learning: they rely entirely on the training samples drawn from the specific sensory domain with which they interact. These samples essentially offer a complete description of the sensory interface, both in terms of its “spatial” and “depth” dimensions, leaving no room for independent exploration. As a simple illustration, if such a system were to encounter a boundary region of the sensory domain that was insufficiently represented or entirely absent in the training data, it may become ineffective, unable to generate meaningful or successful responses (Deng et al., 2023; Gao et al., 2023).

Secondly, it was argued in a number of recent studies that the characteristics of independent sampling and cognitive adaptation can be necessary for evolving learning systems to approach the level of general intelligence that characterizes advanced biological species (Jiang et al., 2022). Fairness of comparison requires then our system to possess such abilities as well.

A system of this class would have the following characteristics.

***Sensory exploration***: The ability to independently traverse, explore, and sample the sensory environment, including the capacity to modify, adapt, and optimize sampling strategies based on internal goals or external feedback.

***Flexible and adaptive cognitive configuration***: The ability to modify, adjust, or optimize cognitive structures and resources such as processing architecture, attention allocation, or memory usage in response to environmental demands or internal states.

***Presence of an evaluative objective***: The existence of a fundamental objective or imperative that is, a criterion against which the system can evaluate and test the effectiveness of its responses to sensory inputs.

Later in the text, intelligent systems that meet these conditions will be referred to as freely-evolving (artificial) intelligence. Based on the preceding arguments, it is conjectured that this class of systems is, at minimum, more likely, and potentially the only type of emergent secondary intelligence that can theoretically reach the level and capabilities associated with general intelligence.

An additional characteristic that we will examine here is a weak constraint on the essential resources of learning (as discussed in Section 3.1):

***Weak constraints on essential cognitive resources, including memory, processing power, energy and others***: This type of artificial intelligent system can be termed as weekly-constrained FEI-W or FEW.

The system can be provided with, and can use some initial saplings of the distribution of sensory stimuli. However, as a distinct characteristic of the statement of the problem as discussed previously, such sampling(s) cannot be expected to be complete, respective the breadth, the entire span of the sensory scope and the depth, the level of detail.

#### 4.2.2 Sensory exploration strategies under weak resource constraints

Sensory exploration in the class of weakly constrained artificial systems presents a distinct set of challenges, shaped not by scarcity of resources but by the absence of internal mechanisms for prioritization and evaluative feedback (Oudeyer and Kaplan, 2007). Unlike biological systems that are evolutionarily adapted to operate under tight resource budgets, weakly constrained systems face the paradox of unconstrained learning: the challenge of making effective decisions in a space where everything is accessible, but nothing is inherently ranked.

To enable quantitative evaluation of sensory exploration strategies in weakly constrained systems, we propose a set of core metrics that reflect both empirical effectiveness and plausibility of realization in cognitive functions. These metrics capture distinct dimensions of learning performance:

- Generality (or the breadth of conceptual span): The extent to which the system produces accurate and context-sensitive responses across the sensory domain.
- Resolution or Robustness (depth of conceptual span): The system’s ability to maintain stable and adaptive behavior in noisy, dynamic, or partially observed environments.
- Prioritization Accuracy and Entropy: The degree to which the system’s sampling strategy aligns with the existential or task relevance of incoming stimuli; entropy of prioritization is a measure of uncertainty in the allocation of attention or sampling effort; lower entropy suggests more decisive and structured prioritization
- Resource Efficiency: The total cognitive and physical cost per unit of effective learning.

These criteria applied to autonomous exploratory learning of the system form the basis for comparative analysis of exploration strategies and support the development of multi-objective optimization frameworks suited to varied environments, tasks, and constraints.

It is instructive to contrast this challenge with biological learning, where constraints serve not merely as limitations but as structuring forces. Human infants, for example, begin with a minimal viable sensorimotor repertoire and gradually expand their cognitive scope through feedback-driven adaptation, guided by relevance and plasticity. In contrast, FEW systems would start with broad, unstructured access to cognitive and sensory resources, but lack intrinsic mechanisms for organizing this access selectively and effectively. This inversion highlights the potential value of biologically inspired scaffolding mechanisms such as curiosity, intrinsic motivation, and attention bottlenecks to enable prioritized and context-sensitive learning in artificial systems.

#### 4.2.3 Sensory prioritization in weakly resource-constrained artificial learning

Not all sensory inputs are equally relevant for the learner’s success. Inputs associated with high existential cost must be prioritized to ensure reliable responses. For weakly resource-constrained artificial systems (FEW), however, the absence of strong intrinsic constraints creates a central difficulty: the system must learn which inputs are critical without relying on fixed external priorities. In dynamic environments, static prioritization is insufficient, requiring adaptive exploration strategies capable of balancing sampling breadth, depth, and risk.

In this section we briefly examine several candidate strategies for sensory exploration in FEW systems. Because FEW architectures remain largely hypothetical and lack standardized implementations or benchmarks, evaluation is necessarily conceptual rather than empirical. Strategies are therefore assessed qualitatively with respect to the dimensions introduced earlier: generality, robustness, prioritization accuracy, entropy of prioritization, and resource efficiency. Given the exploratory scope of the present work, we focus only on a small set of representative strategies that are conceptually compatible with the FEW model.

***Uncertainty-driven prioritized exploration***: Exploration guided by uncertainty directs sampling toward regions where the learner’s internal model is least confident, typically using measures such as prediction error or uncertainty estimates (Deng et al., 2023; Liu and Li, 2023; Sutton and Barto, 2018).

**Summary**: Prioritization accuracy is high because sampling targets expected information gain. Robustness is moderate to high as uncertainty estimates enable refinement of partially learned regions. Generality is moderate since exploration depends on the current internal model and may overlook poorly represented but relevant inputs. Resource efficiency is moderate due to the computational cost of uncertainty estimation.

**Feasibility**: Biological systems; plausible for future FEW architectures.

***Hierarchical and goal-driven exploration***: Hierarchical exploration organizes sensory sampling around goals and subgoals, focusing learning on regions that contribute to task-relevant outcomes (Ott et al., 2022).

**Summary**: Prioritization accuracy and resource efficiency are high due to goal-structured sampling. Generality is moderate to high when goal representations span diverse tasks, but stimuli outside the hierarchy may remain under-sampled. Robustness is moderate to high within structured environments.

**Feasibility**:FEW-class artificial systems; biological systems.

***Evolutionary exploration with sensory feedback***: In feedback-driven exploration, the system gradually adjusts its sampling strategy based on empirical outcomes, reinforcing successful regions while suppressing ineffective or risky behaviors (Koizumi et al., 2024; Nguyen et al., 2022; Pathak et al., 2017).

**Summary**: Robustness is high because behavior is continuously corrected through feedback. Prioritization accuracy improves over time but may initially be limited by sparse signals. Generality is moderate, as early negative feedback may discourage exploration of potentially useful regions. Resource efficiency is moderate due to the need for extended exploration.

**Feasibility**: Biological systems and adaptive artificial systems.

***Developmental (Scaffolded) learning***: Developmental learning expands sensory coverage gradually from a stable core toward more complex regions, mirroring staged cognitive development.

**Summary**: Performance is highly anisotropic: robustness, prioritization accuracy, and resource efficiency are high within scaffolded regions but decline sharply outward. Generality is therefore initially limited but expands as competence grows.

**Feasibility**: Primarily biological; potentially applicable to freely developing artificial systems.

***Constraints-guided exploration***: Constraint-based exploration restricts sampling through explicit or learned safety or value boundaries, preventing exploration of harmful or low-value regions.

**Summary**: Robustness and resource efficiency are high due to reduced exposure to failure states. Prioritization accuracy is high and entropy low because constraints impose strong structure. Generality remains moderate because exploration is restricted.

**Feasibility**: Biological systems and safety or resource-constrained artificial learners.

***Context-adaptive dynamic sampling***: Context-aware exploration adjusts sampling behavior in response to environmental conditions or temporal patterns.

**Summary**: Generality and robustness are high due to situational adaptability. Prioritization accuracy and entropy remain moderate because contextual signals may fluctuate. Resource efficiency is moderate.

**Feasibility**: Biological systems; plausible for advanced artificial learners.

#### 4.2.4 Synthesis

This overview highlights the trade-offs between generality, robustness, prioritization accuracy, and resource efficiency faced by next-phase artificial systems capable of independent empirical exploration. While the reviewed strategies offer different advantages, none resolves the central challenge faced by weakly constrained learners: the absence of intrinsic mechanisms for determining which exploration strategy should govern behavior under changing environmental conditions.

In this sense, the principal limitation of FEW systems is not primarily computational or energetic, but organizational. Unlike biological learners, which operate under strong evolutionary and metabolic constraints that implicitly structure attention and prioritization, weakly constrained systems must operate in environments where virtually all inputs are accessible yet none are inherently ranked. This creates the paradox of unconstrained learning: effective adaptation requires prioritization, but no intrinsic mechanism initially specifies what should be prioritized.

From this perspective, no single exploration strategy dominates across all evaluation dimensions. Rather, effective performance is likely to emerge from adaptive integration: dynamic mechanisms capable of combining the responsiveness of context-sensitive sampling, the precision of goal-driven exploration, and the efficiency of biologically inspired constraints. This observation motivates the concept of a *meta-strategy*: a higher-level regulatory mechanism that enables a system to select, combine, or shift between exploration strategies in response to context, internal state, and empirical feedback.

### 4.3 A quantitative framework for meta-strategic sensory exploration

It has been argued that, in a freely evolving intelligent system, a fixed or rigid sensory exploration strategy may not support optimal empirical performance, particularly when that performance must adapt to changing environments and internal states. From this perspective, an alternative approach emerges: a selective and dynamic choice of strategy from a set of feasible options, guided by empirical success and resource cost. This meta-strategic approach can be formalized as follows:

We define *General sensory traversal* as a meta-strategy: rather than committing to a single sampling method (e.g., entropy-based, curiosity-driven, reinforcement-prioritized etc.), the system can maintain a higher-order policy that selects among different traversal strategies depending on the environment, internal state, or past success. By abstracting the traversal process, the model can emphasize how a system can learn to choose how to learn which mirrors biological flexibility and provides a route toward general intelligence.

It operates within the formal quantitative framework of *adaptive sensory sampling*:

Given the sensory scope (domain) *S*, a traversal process/strategy is defined as a mapping: *T*_k_: *S* → R, which assigns a measure of sampling likelihood or priority to different regions based on a certain criterion. Then the dynamic for the selection of a current strategy can be described by the optimization principle:


$${T_{k}\in T:E\;\;|}_{x∼T_{k}}\;(C(x)-\lambda \cdot R(T_{k}\;))\to \max\;$$ (6)

where:

*C*(*x*): the empirical success or competence.
*R*(*T*_k_): the resource or risk cost of the traversal strategy *T*_k_.
*T*: the space of candidate strategies.
*λ*: the regularization or penalty coefficient (reflecting resource sensitivity or environmental pressure), a Lagrange multiplier.
*E*: the cumulative measure (e.g., a sum or integral).

Equation 6 provides a quantitative foundation for the adaptive, condition- and context-sensitive selection of sensory sampling strategies a formulation we refer to as the Adaptive Sampling Framework (ASF) which will be explored in further detail in future work.

#### 4.3.1 A formalized illustrative model of meta-strategic exploration

To illustrate meta-strategy selection under empirical accountability, consider a freely evolving system with a finite set of exploration strategies T={A,B} , each represented as a functional configuration of sampling heuristics and resource allocation. The system continuously monitors empirical success C(t) , defined as the task-relevant performance measure, and uses it to guide meta-control.

##### 4.3.1.1 Threshold-based exploration

In the simplest regime, a meta-control loop monitors C(t) ; when performance falls below a threshold μ , the system alternates between available exploration strategies. This mechanism enables rapid adaptation to changing conditions without requiring detailed environmental models. It can be effective in simpler interactive environments with a limited set of exploration strategies.

##### 4.3.1.2 Context-sensitive selection

The meta-controller associates strategies with sensory contexts I , preferentially deploying the one maximizing C(t) . Over time, the system updates these mappings, improving efficiency and robustness as environments change:


$$t_{k}\in T:t_{k}\to I,\text{select}\;t_{k}\;\text{maximizing}\;E(C(x)|t_{k}].$$

###### 4.3.1.2.1 Exploratory innovation

If all existing strategies fail ( ∀t∈T:C(t)<μ ), the system generates new candidate strategies $\tilde{t}=\Phi (I,T)$ , iteratively testing them under regime (i) until empirical viability is achieved. Successful strategies expand the available set S ; unsuccessful ones are discarded.

Although simplified, the illustrative model presented below demonstrates that meta-control over exploration strategies can be realized using only empirically accessible success signals, supporting its feasibility in weakly constrained autonomous systems.

Let us consider three essentially different types of environmental configurations:

-
$E_{1}$ : Stable environment
-
$E_{2}$ : A disruptive but partially predictable environmental change
-
$E_{3}$ : A novel environmental regime with no previously known viable strategy
  Initially, under a stable environmental regime $E_{1}$ , the system adopts a depth (fine-grained) focused exploration strategy $s_{1}$ , yielding high and stable empirical success C(t) .

At time $\tau_{1}$ , environmental conditions may shift to $E_{2}$ , resulting in a gradual degradation of C(t) below the viability threshold μ . Meta-control detects this decline and transitions the system to a context-sensitive strategy $s_{2}$ , allowing lateral exploration (within the current resource constraints) and eventual recovery of empirical success by matching behavior to new configurations of environmental states.

At time $\tau_{2}$ , the environment enters a novel regime $E_{3}$ , in which all previously available strategies fail to maintain empirical success. Upon persistent violation of the viability criterion, meta-control escalates to an exploratory regime, generating new candidate strategies. If a viable strategy $s_{\mathrm{new}}$ emerges, the system adopts it, attaining the viability condition C(t)≥μ .

The function of meta-control in this example can be described as:


$$s(t+1)=\{\begin{array}{cc} s(t), & C(t)\geq \mu \\ \arg\max_{s\in S}E[C|s,I(t)], & C(t)<\mu \\ s_{\mathrm{new}}(t)=\Phi (t,I(t)) & \forall s\in S,C_{s}(t)<\mu \end{array}$$

Where *S* is the set of available exploration strategies.

In each of the regimes considered above, maintaining a fixed exploration strategy would lead to inferior adaptation and a progressive deterioration of empirical success C(t) . In a stable environment, the best fixed strategy performs adequately, but it becomes suboptimal once environmental conditions shift. Under disruptive change, strategies that previously ensured stable performance may fail to respond effectively, while in entirely novel regimes no pre-existing strategy may remain viable. The meta-control mechanism addresses this limitation by dynamically preserving, switching, or generating exploration strategies based on empirical performance signals. A compact summary of these regimes and corresponding meta-control actions is provided in Table 1.

**Table 1:** Empirical success-based meta-strategic exploration.

Environment type	Strategy	Empirical success *C*(t)	Meta-control action
E_1_	$S_{1}$	C(t)≥μ (stable)	Hold current strategy
E_2_	$S_{1}$ → $S_{2}$	C(t)<μ (declining → recovery)	Alternate strategies to satisfy empirical criterion
E_3_	$S_{2}$ → $S_{\mathrm{new}}$	S:C(t)<μ (declining → irrecoverable → recovery innovate)	Invent and test new exploration strategies to restore adherence to empirical criterion

The meta-strategy-based operation model presented here illustrate how a meta-strategy layer can support adaptive learning without fixed prioritization rules, while preserving empirical accountability and resource awareness.

#### 4.3.2 Phenomenological and empirical support for meta-controlled exploration

Phenomenological accounts of human and animal learning provide strong intuitive support for the existence of meta-level control over exploratory strategies. Learners do not merely explore within a fixed behavioral or cognitive policy, but routinely shift *how* they explore in response to perceived success, failure, or stagnation. Subjectively, this is often experienced as a transition between focused exploitation, strategic reorientation, and more open-ended or creative modes of inquiry, suggesting the presence of internal signals that monitor learning progress and regulate exploratory style rather than individual actions alone.

Empirically, adaptive systems across biological scales exhibit comparable patterns. Behavioral studies indicate that organisms modulate exploration intensity, risk tolerance, and goal structure based on accumulated feedback, uncertainty, and resource availability. Such modulation operates on slower timescales than moment-to-moment action selection and often governs transitions between qualitatively distinct learning regimes, consistent with a form of meta-control. Importantly, these transitions are not random but appear constrained by prior competence, energetic cost, and anticipated informational gain (Aston-Jones and Cohen, 2005; Hills et al., 2015).

From an artificial systems perspective, meta-controlled exploration aligns with well-established observations that fixed exploration strategies perform poorly in open-ended or weakly constrained environments (Botvinick et al., 2019). Systems that can monitor empirical success and adjust their exploratory policies accordingly demonstrate greater robustness to non-stationarity, improved resource efficiency, and reduced risk of pathological behaviors such as premature convergence or unbounded exploration. Even simple success-triggered switching or strategy matching mechanisms can yield disproportionate gains in adaptability, suggesting that meta-control need not be complex to be effective.

Taken together, these phenomenological and empirical considerations support the plausibility of meta-control as a general organizing principle for exploration in both biological learners and freely evolving artificial systems. In the context of FEW agents, such mechanisms provide a minimal yet powerful means of coordinating learning strategies across developmental stages, environmental regimes, and resource constraints, while remaining compatible with the operational definitions of empirical success introduced earlier.

## 5 Synthesis and discussion

This work presented a theoretical and comparative modeling framework for analyzing the learning dynamics of early-stage biological learners and weakly constrained, next-generation artificial systems. Despite profound differences in resource constraints and architecture, both classes must traverse and internalize complex sensory environments. Our models sought to formalize the distinct strategies each type adopts, naturally or by design under differing cognitive and structural regimes.

Framed by the research questions articulated earlier, this analysis examines how essential cognitive and physical constraints shape the formation and adaptation of learning strategies in different classes of intelligent systems. In this context, biological cognitive systems illustrate how efficient learning can emerge under severe resource limitations through mechanisms such as relevance-based attention, internally regulated feedback, and context-sensitive adaptation. These mechanisms enable natural intelligence to sustain flexibility and robustness despite strict constraints on energy, memory, and processing capacity.

By contrast, contemporary artificial learning systems are typically developed under conditions of relative resource abundance. While such systems achieve strong performance in narrow, well-defined domains, they often lack intrinsic models of constraint, autonomous prioritization, and endogenous feedback regulation. As a consequence, their capacity for robust generalization and adaptive response in novel or uncertain environments remains limited, motivating the comparative modeling approach adopted in this work.

The analysis we attempted in this study supports the view that resource constraints should not be regarded solely as limitations, but as essential conditions that shape intelligent behavior. Across both biological and artificial substrates, cognitive performance can be characterized and analysed along the identified functional dimensions which together expose essential differences between current-stage artificial systems and natural intelligence. By abstracting and functionally incorporating key principles observed in biological cognition, including selective processing, context-sensitive learning, and efficiency-driven adaptation, future artificial systems may achieve higher levels of generalization, autonomy, and resilience.

In the case of early biological learners, we introduced a discrete anisotropic learning model informed by prioritized developmental sensory inputs. It captures the adaptive strategy of prioritizing cognitive depth before expanding conceptual span, reflecting observed progression in perceptual resolution and resilience in infancy. This “onion-shell” model of cognitive expansion rooted in strong local sampling under severe constraints is consistent with developmental neuroscience findings on cortical plasticity and region-specific maturation (Johnson, 2001; Knudsen, 2004), as well as evidence of staged perceptual and motor exploration in infants (Huttenlocher and Dabholkar, 1997).

In contrast, for weakly constrained artificial systems (FEI-W, FEW class), we developed a general quantitative framework for strategy-guided, condition-sensitive sensory sampling. Without natural constraints to enforce structure or priority, such systems risk unfocused exploration and misallocation of attention. Our proposed model supports adaptive strategy selection based on empirical feedback, environmental structure, and empirical success, connecting to frameworks such as constrained optimization, intrinsic motivation, and meta-reinforcement learning (Dolgikh, 2024; Elman, 1993; Beck et al., 2023). These approaches are increasingly explored in AI research aiming to generalize beyond narrow externally-defined tasks.

A comparative analysis of several sensory traversal strategies highlights essential trade-offs. Strategies optimized for broad sensory span may underperform in resilience, while those that prioritize depth may incur slower generalization. This mirrors findings from curriculum learning, active learning, and uncertainty-driven exploration, where no single sampling strategy dominates across environments. Notably, biological systems appear to optimize these trade-offs dynamically through plasticity and embodied interaction (Cangelosi and Schlesinger, 2018), a challenge yet to be fully addressed in artificial learners.

Overall, our analysis suggests that adaptability in general intelligence stems not only from capacity or learning algorithms, but from the co-evolution of structural constraints, environmental interaction, and strategy-level flexibility. This reinforces current efforts in developmental robotics, hierarchical exploration and sensorimotor alignment (Meyer and Wilson, 1991; Oudeyer et al., 2007), and offers a formal foundation for analyzing sampling strategies in emergent intelligent behavior.

A central limitation of the present study is that the proposed models are intentionally theoretical and illustrative, designed to clarify functional trade-offs and constraint-driven dynamics rather than to provide empirically validated performance claims. In particular, key parameters such as learning interval duration, resource increments, and environmental variability are treated abstractly, without calibration to specific biological or artificial implementations. This reflects both the exploratory aim of the framework and the current lack of standardized benchmarks for weakly constrained, autonomously developing systems.

## 6 Conclusion

In this work we examined how essential constraints and empirical accountability shape the organization of exploration and learning in biological and artificial systems. Using the quantifiable factor of empirical success as a common evaluation criterion, we developed a comparative framework that links learning performance to the structural conditions under which exploration occurs.

The presented results address the research questions posed in this study. First, the analysis demonstrates that the nature and severity of resource constraints strongly influence the organization of sensory exploration strategies. Under severe constraints, such as those characterizing biological early development, learning tends to prioritize depth over span, favoring robust representations within a limited sensory domain. The discrete anisotropic learning model introduced here formalizes how such depth-first learning can emerge as an efficient response to limited computational, energetic, and temporal resources. Second, the results clarify how different constraint regimes shape the balance between robustness and conceptual breadth. Strongly constrained learners tend to follow staged, scaffolded trajectories in which competence expands gradually outward from stable core representations, whereas weakly constrained artificial learners face the opposite challenge: excessive freedom of exploration without intrinsic prioritization mechanisms can lead to inefficient sampling and unstable learning dynamics. Finally, the analysis indicates that meta-level regulation of exploration strategies can improve empirical success in weakly constrained systems. The illustrative meta-strategy model shows how monitoring empirical success signals enables a system to preserve effective strategies in stable environments, switch strategies when conditions change, and generate new strategies when previously viable approaches fail, thereby maintaining empirical success where fixed strategies alone would deteriorate.

These results suggest that the effectiveness of learning strategies cannot be understood independently of the constraint regime in which learning occurs. Resource constraints therefore function not merely as limitations but *as structuring forces that shape the trajectory of adaptive intelligence*. From this perspective, differences between biological and artificial learners arise less from their substrates than from the ways in which resource awareness, prioritization mechanisms, and empirical feedback are embedded in their learning architectures.

Several directions for future work follow from this framework. The proposed models can be instantiated in controlled simulations or developmental robotics settings to test how variations in resource budgets, feedback structures, and exploration policies influence empirical success. More broadly, the framework provides a conceptual bridge between developmental neuroscience, machine learning, and complex adaptive systems, offering a shared basis for investigating how intelligent systems can learn efficiently under realistic environmental and resource constraints.

## Data Availability

The original contributions presented in the study are included in the article/Supplementary material, further inquiries can be directed to the corresponding author/s.

## Footnotes

1 For context, it has been estimated that if a human were to process the dataset used to train GPT-3 assuming a few seconds per sentence and no breaks for sleep, meals, or other interruptions it would take well over a century to complete.
