# Supplementary material for: Learning under constraints: a theoretical framework for comparing resource-constrained learning in biological and artificial systems
Source: Front Comput Neurosci. 2026 Jun 17;20:1636604. doi: 10.3389/fncom.2026.1636604 (PMC13318973; doi:10.3389/fncom.2026.1636604)
Supplement: Supplementary file 1 [file Supplementary_file_1.docx]

**Appendix A. Glossary of Common Terms**

1. Sensory scope: The domain or region of the environment from which a learner can receive and process sensory inputs.

2. Empirical success: A measurable criterion of learning effectiveness defined by the uniformity and robustness of successful responses across the sensory scope; operationally described by the system’s sustained ability to maintain functional behavior under environmental variability.

3. Freely-evolving artificial intelligence (FEI): A class of systems that develop autonomously through sensory interaction rather than pre-programmed rules; FEI-W denotes weakly constrained variants with broad exploratory capacity.

4. Adaptive cognitive configuration: The system’s dynamic reorganization of its internal structure to optimize performance under cognitive and physical resource constraints.

5. Anisotropic priority-based exploration: A non-uniform sampling strategy where exploration is directionally biased toward higher-priority stimuli within the sensory scope.

6. Imperative-driven sampling: A sampling approach steered by internal motivational imperatives instead of external rewards or fixed schedules.

7. Utility surface in the sensory scope: a conceptual map indicating where the greatest learning benefit lies within the system’s sensory environment.
