# Supplementary material for: Learning under constraints: a theoretical framework for comparing resource-constrained learning in biological and artificial systems
Source: Front Comput Neurosci. 2026 Jun 17;20:1636604. doi: 10.3389/fncom.2026.1636604 (PMC13318973; doi:10.3389/fncom.2026.1636604)
Supplement: Supplementary file 2 [file Supplementary_file_2.docx]

# **Appendix B. Comparative Analysis of Biological and Artificial Learning**

Comparative analysis of learning characteristics of natural and artificial intelligent systems was conducted along five characteristics (axes) identified in Section 3.4. The interval-scale ranges used in the analysis were: *minimal/vanishing, small, moderate, significant,* and *critical*. The results of the comparative analysis are shown in Table 1.

Table B1. Comparison of Learning Processes in Natural and Artificial Intelligent Systems

| **Characteristic** | **Natural (Biological) Intelligence** | **Artificial Intelligence (Current stage)** | **Artificial Intelligence (Next stage)** |
| --- | --- | --- | --- |
| Resource awareness | Justification: advanced, efficient and integrated sensitivity to energy and resource constraints.  Value: Significant–Critical | Absent or externalized; conventional systems commonly lack integration of resource constraints.  Value: Small | Emergent resource awareness and prioritization of sensory stimuli to shape learning strategy.  Value: Moderate |
| Empirical feedback | Justification: well-developed, based on survival, homeostasis and existential objective.  Value: Critical | External, based on task-specific loss functions and/or performance metrics.  Value: Small–Moderate | Empirical accountability is critical for effective self-guided learning.  Value: Moderate–Significant |
| Structural plasticity | Justification: Continual, context-driven, and attuned to empirical feedback.  Value: Moderate–Significant | Batch-trained, data-driven, commonly static in deployment.  Value: Minimal | Certain structural plasticity necessary for integration of empirical feedback.  Value: Small–Moderate |
| Sampling autonomy | Justification: demonstrate resource-efficient and context-sensitive autonomous sampling strategies.  Value: Significant | Even advanced current stage systems are trained on externally provided data and have minimal sampling autonomy.  Value: Minimal | Sampling autonomy essential for attaining empirical success in self-guided learning.  Value: Moderate |
| Goal definition | Justification: internally-determined, flexible and evolving with sensory context.  Value: Significant–Critical | Externally assigned, generally rigid and immutable.  Value: Small | Emerging goal setting under existential objective.  Value: Moderate. |

Representative literature supporting the assessments in Table B1 includes:

Resource awareness: [1-3,5,11,21,47]; Structural plasticity: [12-14,20-22,28,29]; Empirical feedback: [13,20-23,30,31,45,67,69]; Sampling autonomy: [35,36,42-44,70]; Goad definition and directed behavior: [13,52,65,67,68,71].
