# Supplementary material for: Learning under constraints: a theoretical framework for comparing resource-constrained learning in biological and artificial systems
Source: Front Comput Neurosci. 2026 Jun 17;20:1636604. doi: 10.3389/fncom.2026.1636604 (PMC13318973; doi:10.3389/fncom.2026.1636604)
Supplement: Supplementary file 3 [file Supplementary_file_3.docx]

# **Appendix C. Discrete Interval Simplified Simulation Model**

The simulation model we used is designed to explore the dynamic relationship between two variables that describe learning progress in the discrete model, $y$ (learning depth) and $\phi$ (conceptual span), over a fixed number of iterations.

At each step within an iteration, the increment in $y$ (depth) is calculated as $dy=\sqrt{y}$, while the increment in $\phi$ is determined by $d\phi=\alpha\cdot dy$, where $\alpha$ is the exploration factor that describes the relationship between conceptual depth and span discussed in Section 4.1.2.

The simulation runs for a set number of iterations, each consisting of a fixed number of steps. After each iteration, a new resource increment of $R$ is added, and the process continues. This setup allows us to examine how the exploration "cone" is shaped by the progression of $y$and $\phi$ is and the parameters $\alpha$and $R$.

**Assumptions**

1. The choice of the depth modeling function is a parameter of the model. We assumed that a given conceptual depth facilitates further exploration using more conservative polynomial trend.

2. It was assumed that cognitive characteristics $y$and $\phi$start at zero.

3. Based on the arguments discussed in Section 4.1.2 it was assumed that at the early stage of the process, depth exploration dominates: $\alpha\ll1.$

In the simulation model we used, $R$ is constant, and $\alpha$ is very small due to aforementioned assumption (ranging from 0.001 to 0.01). The simulation was run for 10 iterations, with 5 steps in each iteration. The results showed that while $y$ grows independently of $\alpha$ (the exploration factor) the progression of $\phi$is directly proportional to $\alpha$, leading to a steeper increase in $\phi$ for higher values of $\alpha$. This simple model effectively demonstrates how small changes in the exploration factorcan significantly alter the trajectory of the system.

The results of the simulation are shown in Figure 3.


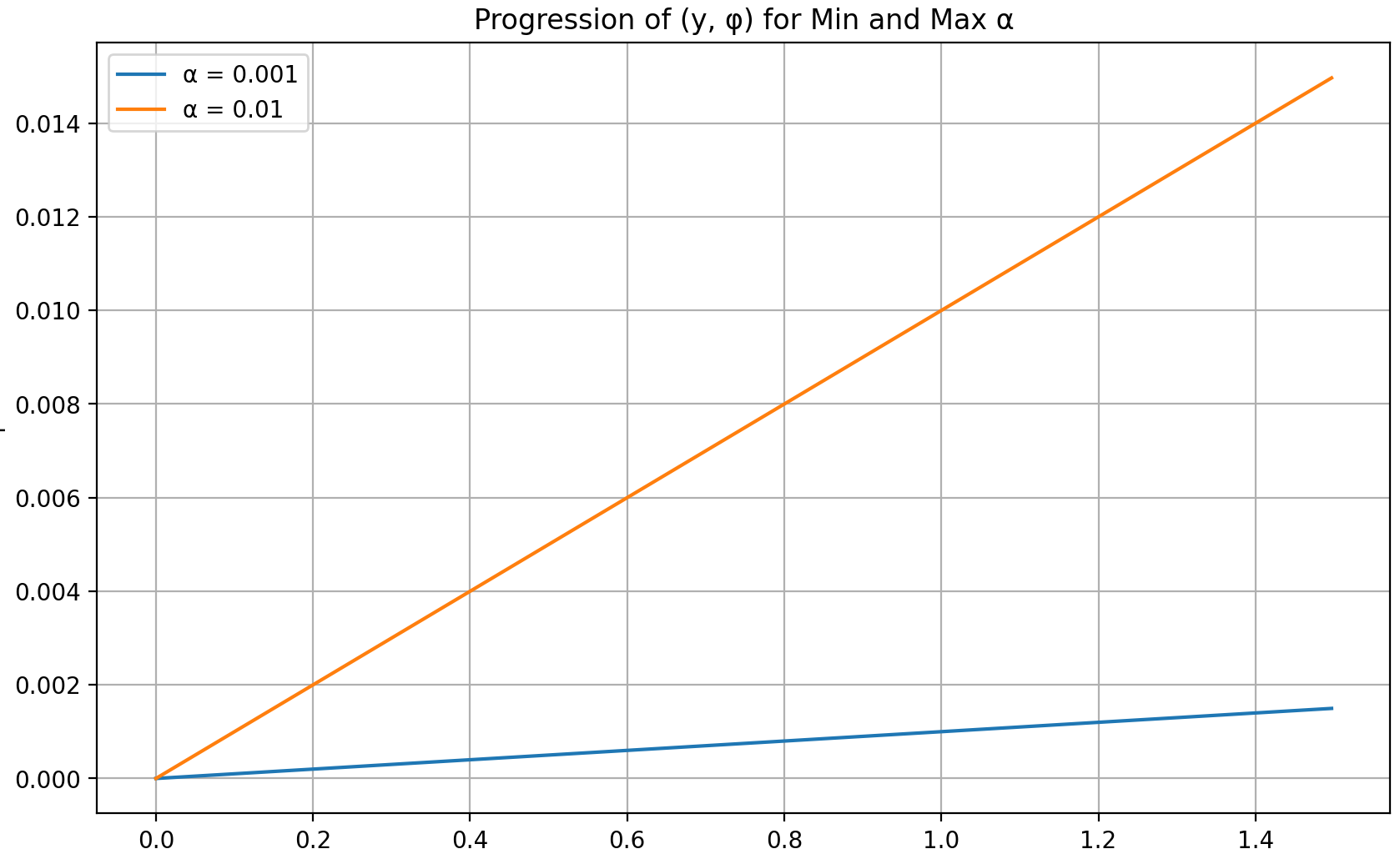


Figure 3. Cognitive exploration region in the discrete interval model.

While the model presented here is intentionally simple, it effectively illustrates the fundamental trade-offs between the factors that shape cognitive exploration. By varying the parameter $\alpha$and observing its impact on the progression of $y$ and $\phi$, we gain valuable insights into how small changes in exploration dynamics can lead to significantly different outcomes. This foundational understanding sets the stage for more detailed and sophisticated models in future studies, where additional parameters, constraints, and real-world complexities can be incorporated to further refine our understanding of cognitive processes.
